# Activated carbon fibers for toxic gas removal based on electrical investigation: Mechanistic study of p-type/n-type junction structures

**DOI:** 10.1038/s41598-019-50707-x

**Published:** 2019-10-08

**Authors:** Byong Chol Bai, Young-Seak Lee, Ji Sun Im

**Affiliations:** 1 Chungnam National University, Institute of Chemical and Biological Engineering, Daejeon, 34134 Republic of Korea; 2 Chungnam National University, Departments of Applied Chemistry and Biological Engineering, Daejeon, 34134 Republic of Korea; 3 Korea Research Institute of Chemical Technology (KRICT), C-Industry Incubation Center, Daejeon, 34114 Republic of Korea; 4 University of Science and Technology (UST), Daejeon, 34113 Republic of Korea

**Keywords:** Atmospheric chemistry, Porous materials

## Abstract

In this study, we evaluated the potential use of CuO-ZnO combination structures with activated carbon fibers (ACFs) for the adsorption (by ACFs) and electrochemical detection (by CuO-ZnO) by of SO_2_ gas. The gas adsorptivity was concluded to improve as a result of the synergetic effects of physical adsorption by the micropores and mesopores, the specific surface area developed by chemical activation and the chemical adsorption reaction between SO_2_ and the transition metals introduced in the CuO-ZnO combination structures. From comparison of the SO_2_ sensing properties, the CuO-ZnO combination structures with ACFs exhibited the fastest sensing capability. This result can be attributed to the larger specific surface area of the semiconductor, which extended its depletion layer by forming p-type CuO/n-type ZnO junctions. This phenomenon led to good SO_2_ detection through a decrease in the resistance; thus, the contributions of the sensing responses of p-type CuO and n-type ZnO represent a predominant characteristic of the sensor. These types of mechanisms were proven through various physicochemical and electrical characterization methods, especially through evaluation of the SO_2_ sensing capability of the CuO-ZnO combination structures with ACFs. The reversible sensing capability indicates that the p-n junction structure changed the electrical properties of the ACFs, leading to an intriguing sensing mechanism.

## Introduction

Unlike soil and water pollution, air pollution is not a local problem limited to the country where the pollution source is located; rather, air pollution is an international issue because it spreads over a wide area, including adjacent countries, due to its characteristics^1^. Major air pollutants include SO_x_, NO_x_, CO, CO_2_, volatile organic compounds (VOCs), and dust^2^. Previously developed materials with porous structures have been extensively used in industrial purification and chemical recovery applications due to their large specific surface areas and pore volumes. In physical adsorption capacity of activated CFs (ACFs) is largely linked to their pore volume, specific surface area and pore size distribution (PSD)^3,4^. The adsorption of polar molecules, such as SO_x_ and NO_x_, can also be strengthened by specific interactions with functional groups on the carbon surface^5–7^. In typical industrial conditions, the SO_2_ adsorption efficiencies primarily depend on the chemical properties of the carbon surface rather than the general textural parameters^8,9^. Additionally, a homogeneous micro-PSD (MPSD) with a mean pore diameter of approximately 7 Å can substantially enhance the SO_2_ adsorption capacity^10,11^. Therefore, controlling the physical and chemical properties and determining the best combination of these properties are both important for creating ACFs with good adsorption properties. To further improve the gas adsorption properties of such ACFs, studies on strategies for enhancing the adsorption efficiency and catalytic characteristics have been conducted by adding functional groups to the surfaces of ACFs or impregnating the fibers with a transition metal oxide.

In recent decades, SO_x_ trapping has been investigated in many studies using a wide variety of metal structures, such as Cu, Ag, Ni, Fe, Co, and Pt. Cu has been utilized in many studies because it is inexpensive and exhibits excellent reactivity^12–14^. Moreover, in which two different metal oxides structures have recently become attractive candidates as sensing layers due to their potential to enhance gas sensitivity by a larger modulation in current through the p-n junction barrier than a single metal structure^15,16^.

Therefore, these kinds of material should satisfy three main criteria when it needs combination each other. First, the sensor should be useful at room temperature; second, it has to be stable under harsh conditions, such as acidic environments and high temperatures; and finally, it should be highly sensitive to toxic gases. Over the last several decades, ACFs have attracted attention as a promising material that could satisfy the above criteria because ACFs have a high specific surface area for enhanced gas adsorption, excellent electrical properties for high sensitivity and chemical stability for resistance to acidic and high temperature conditions^17–19^. The bottleneck that limits the use of ACFs as a gas sensing material is their insufficient sensitivity to low concentrations of target gases.

So, in this paper, we focused on evaluating the potential use of the prepared ACFs for adsorption and electrochemical detection of SO_2_ gas. Here, changes in the microtextural properties were caused by pyrolysis of lyocell fibers followed by chemical activation. Also, the roles of various metal oxide dopants, such as zinc oxides and copper oxides, on ACFs in SO_2_ gas adsorption were investigated. The chemical and textural properties of these carbon materials were evaluated for their potential application in the adsorption of harmful gas.

## Results

### Surface morphology

**Figure 1:**
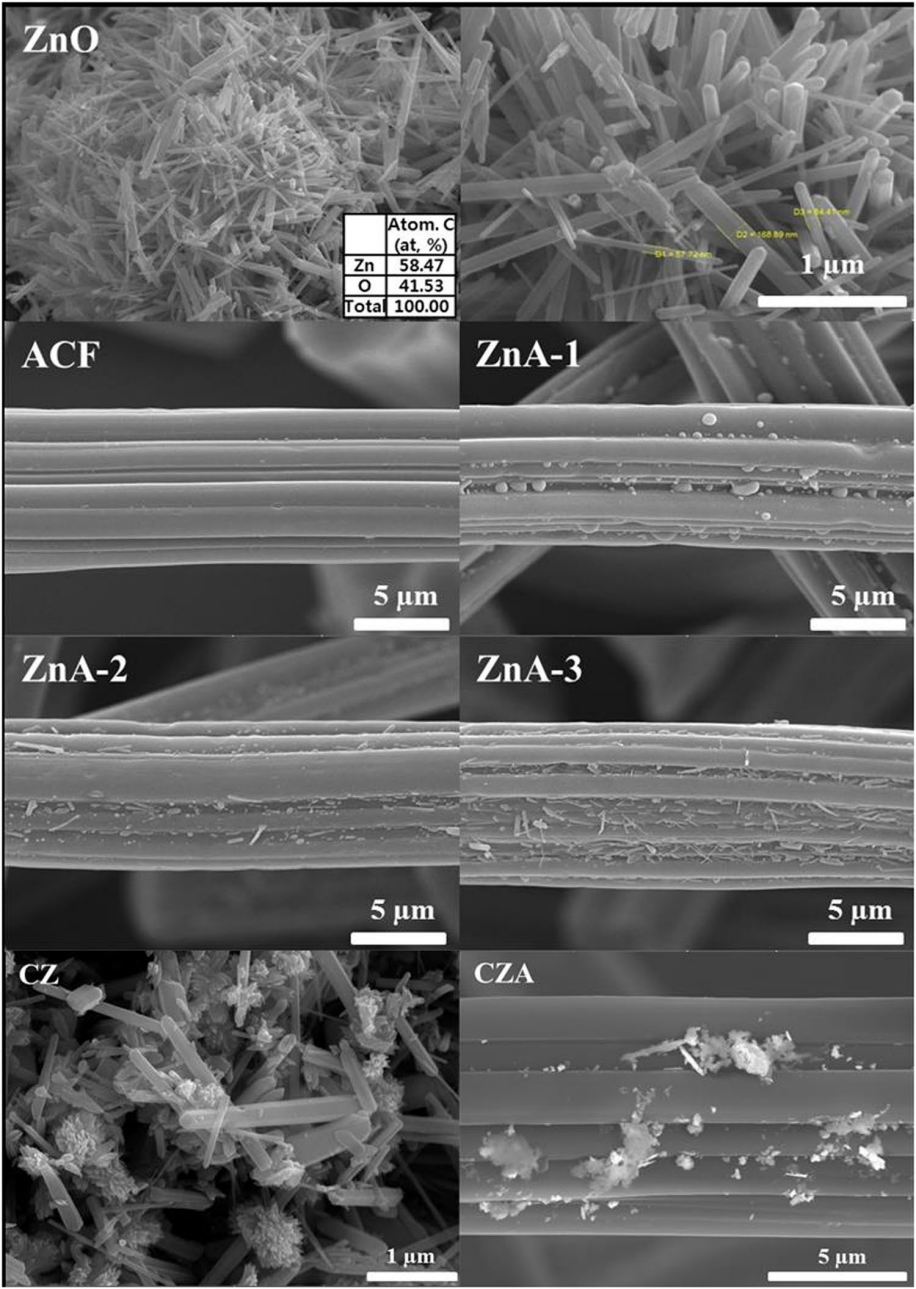
FE-SEM images of ZnO, ZnO-doped ACFs and CuO-ZnO combination with ACFs.

Figure 1 shows SEM images of the surface morphologies of the ZnO particles and ZnO/ACFs (ZnA samples) as a function of the ZnO concentration. Flower-shaped ZnO structures were formed, and the ZnO particles were 50~170 nm in diameter. According to the EDS analysis, the Zn and O contents in the ZnO particles were 58.47 and 41.53%, respectively. The ZnO particles were dispersed over the fiber surface during the preparation of the ZnA sample. Additionally, at low concentrations, the shapes of the ZnO particles were not conducive to growth, and the ability of the ACFs to grow into hexagonal pole shapes was inhibited. SEM images of the CuO/ZnO (CZ sample) and CuO/ZnO/ACFs (CZA sample) samples are shown in Fig. 1. The CuO and ZnO particles aggregated in the CZ sample, and these aggregates dispersed over the fiber surface in the CZA sample. The SEM images revealed that some of the ACF pores were blocked by the CuO and ZnO particles.

### Textural properties

The nitrogen adsorption isotherms of the various metal oxide-doped ACFs are presented in Fig. S1. Additionally, a summary of the textural properties of the ACFs samples is presented in Table 1. The pore textures of the ACFs after treatment by the hydrothermal method were investigated by nitrogen adsorption analysis at −196 °C. As seen in Fig. S1, the adsorption isotherms of the raw and CuA-3 samples showed matched well to Type I adsorption characteristics, indicative of a porous material with micropore diameters smaller than 2 nm^20^. However, the ZnA-3 and CZA samples show a slightly gentler isotherm curve than the others. Mesopores developed between 0.01 and 0.4 P/P_0_, and a hysteresis phenomenon was observed, especially in the ZnA-3 sample, due to aggregation of the ZnO particles during the preparation process, which blocked the pore structures.

**Table 1:** Surface properties of the various metal oxide-doped ACFs.

		Raw	ZnA-1	ZnA-2	ZnA-3	*CuA-3	CZA
BET (specific surface area)	m^2^/g	925	828	822	749	874	718
V_micro_	cm^3^/g	0.365	0.329	0.321	0.304	0.361	0.289
V_meso_	cm^3^/g	0.027	0.044	0.061	0.034	0.027	0.041
V_total_	cm^3^/g	0.392	0.373	0.382	0.338	0.388	0.330

*CuO-sputtered ACFs (Ref. Bai. BC, etc, Materials Chemistry and Physics 200 (2017) 361–367).

As shown in Table 1, the BET specific surface area and pore volume decreased after treatment by the hydrothermal method. The raw sample showed the highest BET specific surface area, whereas the BET specific surface areas of the metal oxide-doped samples were lower. In particular, the specific surface area of ZnA-3 decreased from approximately 925 m^2^/g to 749 m^2^/g relative to that of the raw sample. The pore volume also decreased after treatment by the hydrothermal method. For example, the CZA sample exhibited a total pore volume of 0.289 cm^3^/g, almost 0.08 cm^3^/g lower than that of the raw sample.

### Surface properties of the CuO-ZnO-doped ACFs

**Figure 2:**
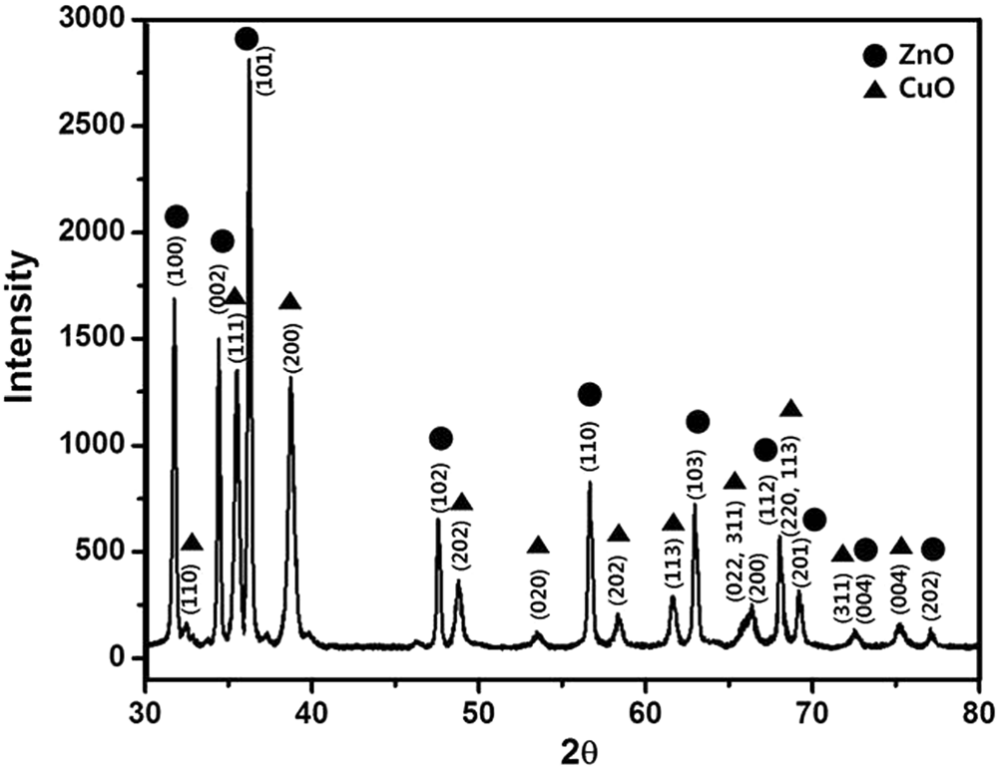
XRD data for the CZ sample (CuO-ZnO combination structure).

The CuO-ZnO combined sample was characterized using XRD, as shown in Fig. 2. The ZnO flowers exhibited main diffraction peaks corresponding to the (100), (002), and (101) planes; these are the general peaks of the hexagonal wurtzite phase of ZnO. In the CZ samples, the XRD patterns matched the monoclinic phase of CuO and the hexagonal wurtzite phase of ZnO. No secondary phases were detected. The XRD peaks of CuO and ZnO in the CZ samples became narrower and more intense due to the hydrothermal treatment.

Figure S2 shows the mapping images of the CZA sample obtained from EDS analysis. As shown by the EDS scan, no intermixing or transformations occurred between the CuO and ZnO particles. The two types of oxides were confirmed to aggregate together and well disperse on the ACF surface. As shown in Table S1, the amounts of CuO and ZnO introduced onto the ACF samples were confirmed to be 8.28% of Cu and 5.33% of Zn by ICP-MS. Based on these results, some of the ACF pores could blocked by the CuO and ZnO particles, resulting in a decrease in the specific surface area. According to comparison of the pore volumes provided in Table 1, the pores were blocked by the metal oxide particles, whereas mid-sized to large pores formed due to the aggregation of CuO and ZnO.

### SO_2_ adsorption properties

To analyse the adsorption of SO_2_ in the fixed bed reactor, breakthrough and saturation curves (C_t_/C_0_ vs. time) were generated, and the data were evaluated using the following equation^21^:

effluent volume:

$${{\rm{V}}}_{{\rm{eff}}}={\rm{Q}}\cdot {{\rm{t}}}_{{\rm{total}}},$$

where Q is the volumetric flow rate (mL/min), and t_total_ is the time of SO_2_ adsorption.

The total uptake capacity (q_total_, mg) is given by the following equation^22^:

$${q}_{total}=\frac{Q}{1000}\,{\int }_{t=0}^{t={t}_{total}}\,C\,dt$$

where C is the SO_2_ concentration at each point.

The total SO_2_ (m_total_, mg) sent to the reactor and the total SO_2_ removed (R_total_, %) were calculated using the following equation^23^:

$${m}_{total}=\frac{{C}_{0}{V}_{eff}}{1000}$$

$${R}_{total}=\frac{{q}_{total}}{{m}_{total}}\times 100$$

The length of the mass transfer zone (MTZ), which refers to the length of the bed over which the concentration in the fluid phase changes from an upper value to a lower value, was computed from the breakthrough curve as follows^24^:

$${Z}_{m}=Z(1-\frac{{t}_{0.05}}{{t}_{0.95}})$$

where Z_m_ is the length of the MTZ (cm), Z is the bed height (cm), t_0.05_ is the time at breakthrough (min), and t_0.95_ is the time at which saturation is reached (min).

The bed utilization of the fixed bed reactor, *f*, is given by the following equation^24^:

$$f=(1-\frac{0.5\times MTZ}{bed\,height})\times 100$$

We studied the adsorption of SO_2_ in a fixed bed reactor packed with the CuO-ZnO combined ACFs to predict the breakthrough and saturation curves. The values were obtained using the formulas above, and the results are shown in Table 2. One of the main advantages of this model is its ability to predict the breakthrough time (t_b_) and saturation time (t_s_), the latter of which was arbitrarily defined as the time corresponding to C/C_0_ = 5%. Compared with the raw sample, the breakthrough and saturation times of the CZA sample increased from 4.74 to 8.79 min and from 14.65 to 22.02 min, respectively. These results show that SO_2_ adsorption is due to the combination of the micropores and the ionic interactions imparted by the introduced metal oxide support. The percentage of SO_2_ removal and adsorption capacity were calculated from the breakthrough curves and are presented in Table 2. The SO_2_ adsorption capacity increased in the following order: raw < ZnA-3 < CZA < CuA-3. These results may be attributed to physical adsorption by the micropores and mesopores. Second, the chemical adsorption of SO_2_ was made possible by the metal oxide support impregnated into the ACF surface. However, the ZnA-3 sample showed a slightly higher SO_2_ adsorption capacity than that of the raw sample. These results show that SO_2_ adsorption occurred through weak ionic interactions with the ZnO support rather than with the CuO support. The bed utilization factors (*f*) were calculated from the length of the MTZ, and the CZA sample had an *f* value of 69.96%, higher than those of the other samples. These phenomena could be explained by the specific surface area and catalyst support because the large specific surface area seemed to interact strongly with the SO_2_ molecules and exert a greater influence than the catalytic effects did. Therefore, the adsorption efficiency of SO_2_ should be investigated according to the specific surface area of the ACFs.

**Table 2:** Parameters obtained from the breakthrough curves of SO_2_ adsorption.

Sample	Superficial Velocity	Initial Concentration	Bed Height	Total Time	Breakthrough Time	Stoichiometric Breakthrough Time	Saturation Time	Effluent Volume	Total Amount Removed	Total Removal	Adsorption Capacity	Length of MTZ	Bed Utilization
	Q	C_0_	Z	t_total_	t_0.05_	t_0.5_	t_0.95_	V_eff_	m_total_	R_total_	q_total_	L_MTZ_	*f*
	(cc/min)	(mg/L)	(cm)	(min)	(min)	(min)	(min)	(cc)	(mg)	(%)	(mg)	(cm)	(%)
Raw	1,500	40.00	2	35.02	4.74	7.92	14.65	52,533	2101.32	28.82	605.51	1.35	66.18
*CuA-3	1,500	40.00	2	43.60	10.21	13.38	27.65	65,400	2616.00	47.72	1248.30	1.26	68.46
ZnA-3	1,500	40.00	2	32.77	3.90	8.02	18.89	49,155	1966.20	31.12	611.88	1.59	60.32
CZA	1,500	40.00	2	39.89	8.79	10.83	22.02	59,835	2393.4	43.40	1038.74	1.20	69.96

*CuO-sputtered ACFs (Ref. Bai. BC, etc, Materials Chemistry and Physics 200 (2017) 361–367).

### Comparison of SO_2_ adsorption to the BET surface area

**Figure 3:**
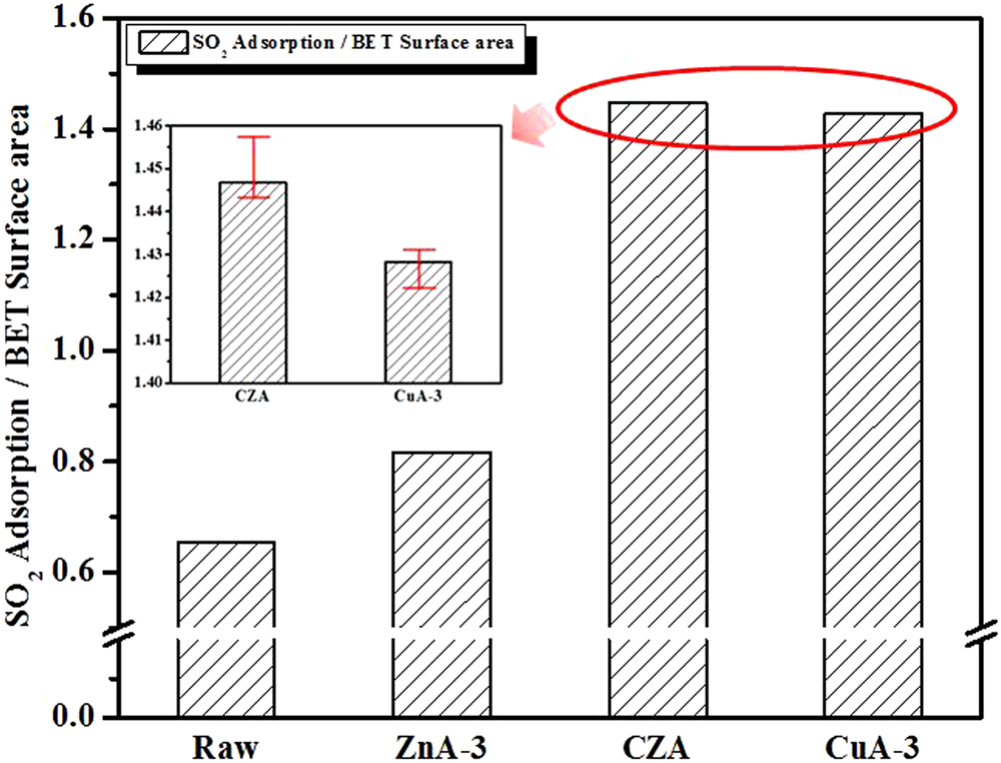
Comparison of the SO_2_ adsorption capacities according to the BET specific surface area of the various metal oxide-doped ACFs.

We examined the chemical effects induced by the enhanced concentration of the various catalyst supports on SO_2_ adsorption to the ACFs and observed an evident correlation between the SO_2_ capacity and the surface properties. Figure 3 presents the SO_2_ adsorption capacity according to the BET specific surface area for the treated ACFs. An increase in SO_2_ adsorption was observed for the ACFs impregnated with the catalyst support. The SO_2_ adsorption capacity according to the BET specific surface area increased in the order of raw < ZnA-3 < CuA-3 < CZA. These results can be explained by the pore structure and EDS data. The textural properties of the ACFs did not exhibit large structural defects after the surface treatment. In particular, the CZA sample exhibited the highest values among all the samples tested. Based on these results, the SO_2_ molecules near the pores were concluded to be more strongly effected than the other molecules with a lone pair of electrons by interactions with the CuO-ZnO combination structure, generating attractive forces that effected the electrons in the SO_2_ molecules. These differences in the surface chemical composition are expected to affect the electrochemical properties, as discussed in next section.

### Mechanistic study of p-type/n-type junction structure

**Figure 4:**
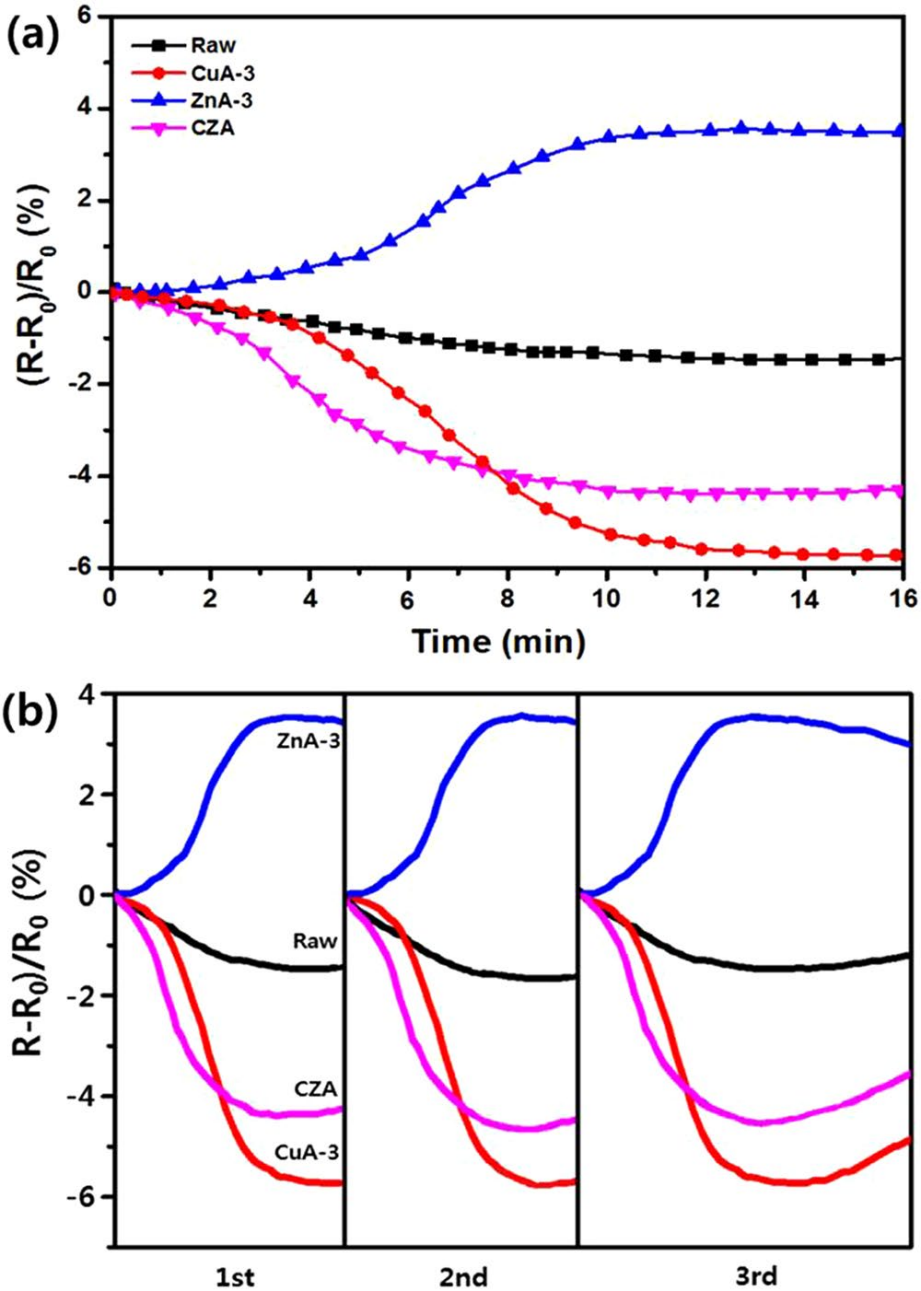
Repeatability of the resistive responses of the various metal oxide-doped ACF gas sensors to repeated SO_2_ exposure.

The resistive response is plotted versus the time of exposure to SO_2_ in Fig. 4(a) in terms of (*R* − *R*_*0*_)/*R*_*0*_, where *R* is the measured resistance and *R*_*0*_ is the initial resistance. The raw sample showed a resistive response of approximately 1% due to the inactive SO_2_ adsorption sites and the electron hopping effect in the narrow carbon pores^25^. The development of a porous structure and a ZnO support enhanced the SO_2_ sensing ability by more than 4%, as shown for ZnA-3. This result is explained by the n-type semiconductor properties of ZnO: the resistance increases with the adsorption of SO_2_, which has remaining electrons. Sample CuA-3 exhibited a high gas sensitivity due to the development of abundant copper active sites for SO_2_ adsorption. The electrical resistance easily changed, and these phenomena could be explained by the pore effect and adsorbed SO_2_ molecules. Conversely, the CZA sample exhibited a fast sensing capability that can be attributed to the larger specific surface area of the semiconductor, which extends its depletion layer by forming p-type CuO/n-type ZnO junctions^26,27^. The microstructural evolution and gas-sensing properties of the CuO particles on the micron-scale ZnO (CuO/ZnO) structures synthesized via a hydrothermal method were studied by Ran Yoo and co-workers^28^. In that report, CuO-doped ZnO-based sensors showed improved sensing capabilities for dimethyl methylphosphonate (DMMP) gas detection. This increased sensing capability could lead to decreased sensing recovery and response times because of an increase in the number of O_2_ vacancies and surface reactions resulting from modification of the surface via CuO doping. Additionally, perfectly reproducible SO_2_ sensing properties with the expected patterns were observed. Figure 4(b) presents the reproducibility results of the samples as SO_2_ sensors. For these measurements, all samples were tested three times by removing the adsorbed SO_2_ gases via heating to 150 °C at a pressure of 1 × 10^−3^ Torr for 10 min. The results revealed almost perfect reproducibility, which can be attributed to the good desorption properties of the samples during the recovery process. This property is a result of the general advantages of porous carbon materials such as activated carbon and ACFs^29,30^.

**Figure 5:**
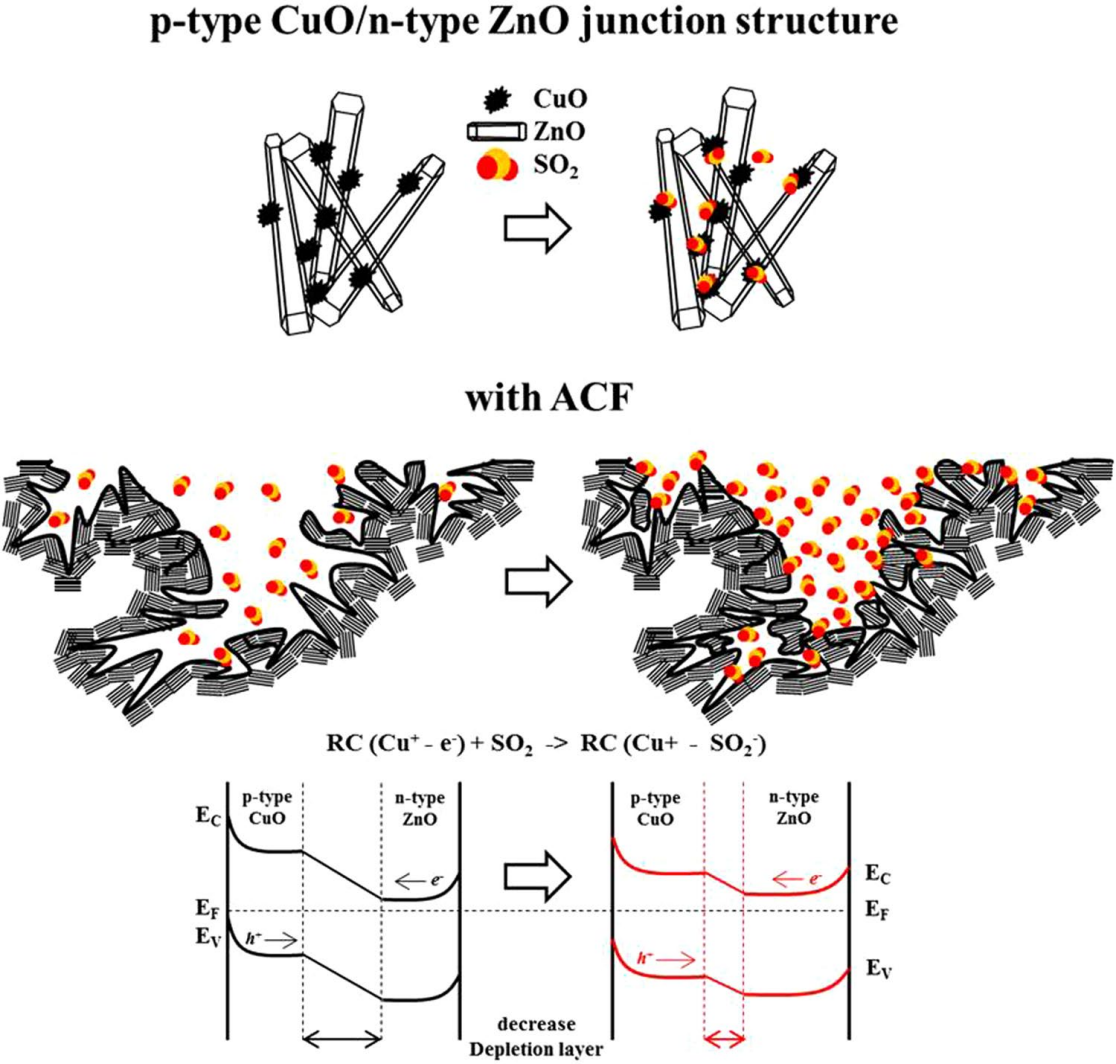
p-CuO/n-ZnO junction structures and their mechanism.

Figure 5 displays a possible mechanism for SO_2_ detection by the CuO-ZnO combination structures with ACFs. In this figure, CuO particles are randomly dispersed on the ZnO flower surfaces. When the CuO particles combine with the ZnO flowers, a p/n junction is formed at the interface between the p-type CuO particles and the n-type ZnO flowers. In this mechanism, electrons transfer from the n-type ZnO to the p-type CuO, and the holes go through the p-type CuO to the n-type ZnO until a uniform Fermi level is established in the system, which leads to band bending in the depletion layer. However, when the CuO-ZnO combination structures are exposed to SO_2_, electrons are released through a gas-sensing reaction between Cu^+^ and negatively charged SO_2_^−^ ions. The reduced SO_2_ molecules combine with the holes in the CuO, decreasing both the depletion layer and the resistance^28^. This phenomenon leads to good SO_2_ detection through a decrease in the resistance; thus, the contributions of the sensing responses of the p-type CuO and n-type ZnO represent a predominant characteristic of the sensor, and the specific surface area of the ACFs plays an important role in the SO_2_ sensing properties.

## Discussion

In this work, CuO-ZnO combination structures were uniformly grown and aggregated on ACFs using a hydrothermal process. The dispersion and aggregation of CuO and ZnO strongly influenced the morphology of the ACFs. The mechanism of pore structure development was altered in the ACFs impregnated with the CuO-ZnO combination structure. The formation of metal oxides was confirmed by XRD analysis, which revealed that the layers were quite uniform and clearly crystallized as CuO and ZnO. EDS analysis indicated that the CuO-ZnO combined ACFs possessed the monoclinic phase of CuO and the hexagonal wurtzite phase of ZnO according to the XRD analysis. No intermixing or identity transformations were observed between the CuO and ZnO particles, but the particles did aggregate and were dispersed on the ACF surfaces. Some ACF pores were blocked by the CuO and ZnO particles, resulting in a decrease in the surface area. Additionally, the pores could be selectively controlled, and a high specific surface area could be obtained by controlling the optimum process variables for introducing CuO and ZnO particles.

In the examination of the SO_2_ adsorption properties, the CZA sample showed a higher capacity for SO_2_ uptake than the other samples. This phenomenon could be explained by the high SO_2_ adsorption capability of the CuO-ZnO-combined ACFs caused by the strong interactions of the CuO-ZnO combination structure with SO_2_. These results demonstrate that the adsorption of SO_2_ is due to the combination of the micropores in the ACFs and the ionic interactions of the introduced metal oxide support. However, the ZnA-3 sample had a small SO_2_ adsorption capacity because SO_2_ adsorption occurred via weak ionic interactions with ZnO. The polarized SO_2_ molecules changed the electrical resistance of the ACFs, resulting in good reproducibility and repeatability as a gas sensor. The CZA sample exhibited the most rapid sensing capability, which can be attributed to the extension of the semiconductor depletion layer by forming p-type CuO/n-type ZnO junctions. When the CuO-ZnO combination structures adsorbed SO_2_, the electrons of SO_2_^−^ were released through the gas-sensing reaction with Cu^+^.

In conclusion, the p-n junction structures facilitated the highly sensitive detection of SO_2_ and decreased the resistance at room temperature. The specific surface areas of the ACFs also played critical roles in improving the SO_2_ sensing properties. This type of investigation is important for testing the performance of gas adsorbents and sensors in real environments. We focused on evaluating the chemical properties of the prepared ACFs and their potential use for SO_2_ adsorption. Because adsorption is the basis for gas removal and sensing, these materials can contribute to protection against toxic gases.

## Methods

### Materials

Lyocell fibers (Kolon Industries, Republic of Korea) were cut into 20 cm lengths (approximately 10 g). Diammonium hydrogen phosphate (DAHP, Sigma-Aldrich Co., USA) was used to enhance the yield of the lyocell fibers during carbonization. Potassium hydroxide (KOH, >98%, Samchun Co., Republic of Korea) was used as a chemical activation agent to develop the pore structure. The process was described in detail in our previous study^31^. Zinc acetate dihydrate (Zn(CH_3_COO)_2_·2H_2_O, >99.999%, Sigma-Aldrich, USA) and sodium hydroxide (NaOH, >98%, Samchun Co., Republic of Korea) were used as reagents for preparing zinc oxide (ZnO) on the ACF surface. Additionally, copper (II) nitrate trihydrate (Cu(NO_3_)_2_·3H_2_O, >99.999%, Sigma-Aldrich, USA) was used as a chemical combination agent for the development of ZnO with copper oxides (CuO).

### DAHP treatment of lyocell

DAHP was used for the chemical treatment of the lyocell fibers to enhance the carbonization yield. First, 10 g of raw lyocell fibers was immersed in 100 ml of 1, 3, 5 and 7 wt% DAHP solutions for 30 min at 60 °C. The excess solution was removed by centrifugation, and the samples were dried overnight at 70 °C under vacuum. The optimal chemical treatment conditions (5 wt% DAHP solution exhibited the best yield enhancement of the lyocell fibers) were determined based on our previous results^32^.

### Preparation of ACFs

Prior to carbonization, the lyocell fibers were stabilized at 300 °C for 30 min in air. The stabilized lyocell fibers were heat-treated in a nitrogen atmosphere at 1000 °C under the following conditions: a heating rate of 10 °C/min, a holding time of 1 h and a nitrogen feed rate of 100 ml/h. The samples that were subjected to the heat treatment are referred to as CFs.

A 6 M KOH solution was prepared as the chemical activation agent based on our previous work^33^. The CFs were placed in an aluminium boat within a reactor. Then, the KOH solution was added at a concentration of 15 ml/g, and the CFs were activated at 750 °C for 3 h in a nitrogen atmosphere. The heating rate was 5 °C/min, and the nitrogen feed rate was 100 ml/min. After the reaction, the samples were washed several times with distilled water to remove residual potassium and dried for 24 h in a 120 °C oven^34^. The resulting sample was denoted LA (lyocell-based ACFs).

### Hydrothermal method

Hydrothermal treatment is the simplest method for generating a relatively uniform dispersion of metal oxides on the ACF surface. ZnO was prepared from 100 ml of 100, 250, and 500 mmol zinc acetate dihydrate solutions and 100 ml of 100, 250, and 500 mmol sodium hydroxide solutions; both types of solution were prepared in distilled water under constant stirring and transferred to an autoclave with the ACFs. The autoclave was heated at 120 °C for 12 h. The ZnO-treated ACFs were collected by centrifugation and washed several times with methanol. After washing, the ACFs were dried at 80 °C for 12 h in air and annealed at 400 °C for 2 h in air. The amount of ZnO that impregnated into the ACFs was quantified using an Inductively coupled plasma mass spectrometry (ICP-MS, Thermo Fisher Scientific iCAP Qc, in Korea Research Institute of Chemical Technology (KRICT)). The manufactured samples are shown in Table S1 and are referred to as ZnA-1, ZnA-2, and ZnA-3 based on the zinc acetate dihydrate concentrations. The ICP-MS results confirmed that 1.34, 3.02, and 6.47 wt% of Zn, respectively, was introduced into the samples.

Furthermore, the CuO-ZnO combined ACFs were prepared from 100 ml of a 500 mmol zinc acetate dihydrate solution and 100 ml of a 500 mmol sodium hydroxide solution; both solutions were prepared in distilled water under constant stirring and transferred to an autoclave with the ACFs. After that, copper (II) nitrate trihydrate (50 mmol dissolved in 100 ml of distilled water) was added under constant stirring for 2 h. After this period, the samples were collected by centrifugation and dried at 80 °C for 12 h in air.

### Physicochemical characterization

To observe the surface characteristics and microstructures of the manufactured samples, field emission scanning electron microscopy (FE-SEM, Hitachi, S-5500, in Korea Basic Science Institute (KBSI) Jeonju Center) coupled with energy dispersive spectrometry (EDS, Quantax 200, Bruker, in KBSI) was used. The textural properties of the prepared ACFs were investigated. The ACF samples were heated under vacuum at 150 °C for 8 h to remove water and some of the impurities adsorbed to the samples prior to analysis; the same preparation method was used for the Brunauer–Emmett–Teller (BET) analysis. We chose this strategy because we thought it was important to achieve the same conditions prior to conducting the analysis. The pore structures of the samples were confirmed by N_2_ adsorption at −196 °C using an ASAP2020 instrument (Micromeritics, in KRICT). The specific surface areas of the samples were determined using the BET equations. The PSDs of the samples were determined using the Horvath–Kawazoe (HK) and Barrett–Joyner–Halenda (BJH) methods. The crystallinity and structural properties of the fabricated photocatalysts were characterized using an X-ray diffractometer (XRD, D8 DISCOVER, Bruker AXS, in KRICT) equipped with Cu Ka radiation.

In this study, we used the fixed bed reactor for the SO_2_ adsorption experiments. In these experiments, 0.1 g of ACFs was placed in a vertical stainless-steel reactor (internal diameter: 5 mm). A thermocouple was placed 1 mm above the ACF sample to measure the internal bed temperature. The flow was maintained at 1500 cc/min, and the SO_2_ concentration was approximately 40 ppm. The analyser continuously monitored the weight gain, and the outlet SO_2_ concentrations were continuously measured using a Teledyne model 7600 device (Teledyne Co., in KRICT). The outlet gas composition was recorded every second.

The effects on the sensitivity and response time of the SO_2_ sensor prepared using the CuO-ZnO-doped ACFs were investigated. The ACF samples (0.1 g) were ground using a mortar and dispersed in 5 g of N, N-dimethylformamide (DMF, Sigma-Aldrich, USA). To disperse ACFs in DMF solution, we used sonicator for 30 min to uniformly disperse the samples in DMF. After sonication, the ACF solution (0.01 g) was dropped onto the silicon wafer (0.5 × 0.5 cm^2^) using a micropipette (Ovation pipette, Vistalab) and spin-coated (ACE-200 spin-coater) at 900 rpm for 4 min. The spin-coated wafer was heated on a hotplate at 50 °C for 10 min to evaporate the DMF. The electrical resistance was measured using a programmable electrometer (Keithley 6514, in KRICT) to evaluate the gas-sensing properties of the prepared sample. This measurement was performed in a 1000-cm^3^ stainless steel chamber. The chamber was connected to gas cylinders (SO_2_ and N_2_), and the gas flow was controlled using a mass flow controller. The prepared gas sensor sample was placed in a sealed chamber under vacuum at 1 × 10^−3^ Torr. Nitrogen gas was injected into the chamber to stabilize the electrical resistance. Then, 40 ppm of SO_2_ (N_2_ balance) was injected into the chamber. The total flow rate of the gas was maintained at 500 cc/min. The change in the electrical resistance was measured at 25 ± 1 °C.

## Supplementary information

Supplementary Information
