# Supplementary material for: Activated carbon fibers for toxic gas removal based on electrical investigation: Mechanistic study of p-type/n-type junction structures
Source: Sci Rep. 2019 Oct 8;9:14458. doi: 10.1038/s41598-019-50707-x (PMC6783449; doi:10.1038/s41598-019-50707-x)
Supplement: Supplementary file 1 — Supplementary Information [file 41598_2019_50707_MOESM1_ESM.doc]

[**Supplementary information**](http://www.nature.com/nmat/journal/v8/n7/suppinfo/nmat2469_S1.html)

**Activated carbon fibers for toxic gas removal based on electrical investigation: Mechanistic study of p-type/n-type junction structures**

**Byong Chol Bai^1^, Young-Seak Lee^1,2^, Ji Sun Im^3,4,*^**

^1^Chungnam National University, Institute of Chemical and Biological Engineering, Daejeon, 34134, Republic of Korea

^2^Chungnam National University, Departments of Applied Chemistry and Biological Engineering, Daejeon, 34134, Republic of Korea

^3^Korea Research Institute of Chemical Technology (KRICT), C-Industry Incubation Center, Daejeon, 34114, Republic of Korea

^4^University of Science and Technology (UST), Daejeon, 34113, Republic of Korea

*jsim@krict.re.kr


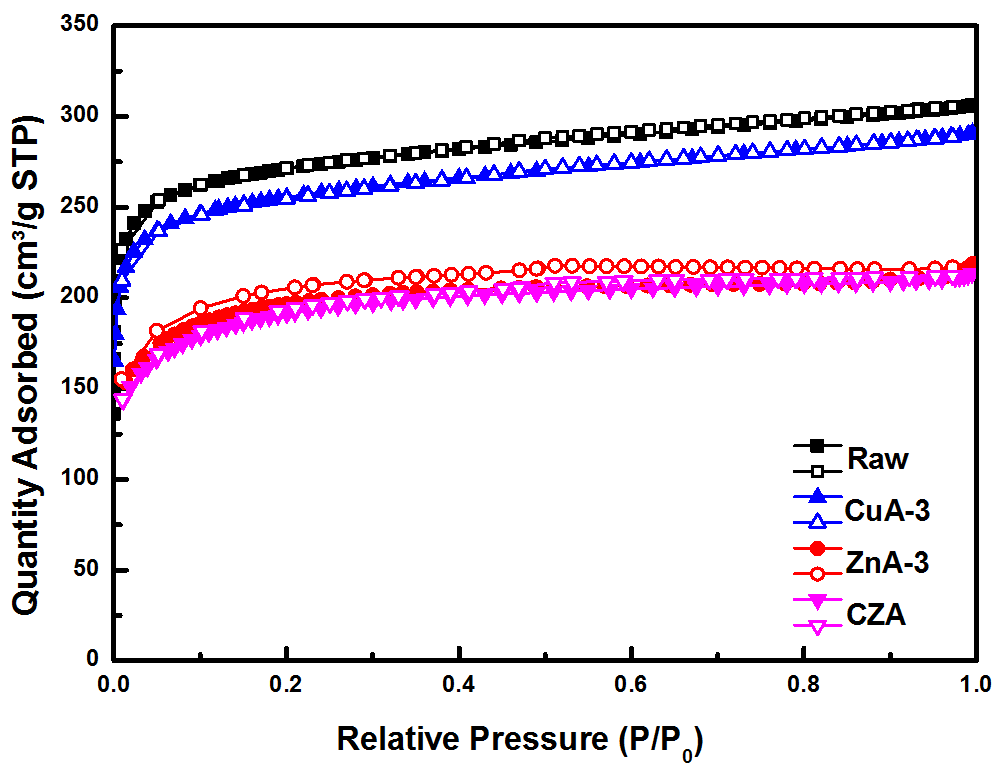


**Figure S1 |** Nitrogen isotherms of the various metal oxide-doped ACFs.


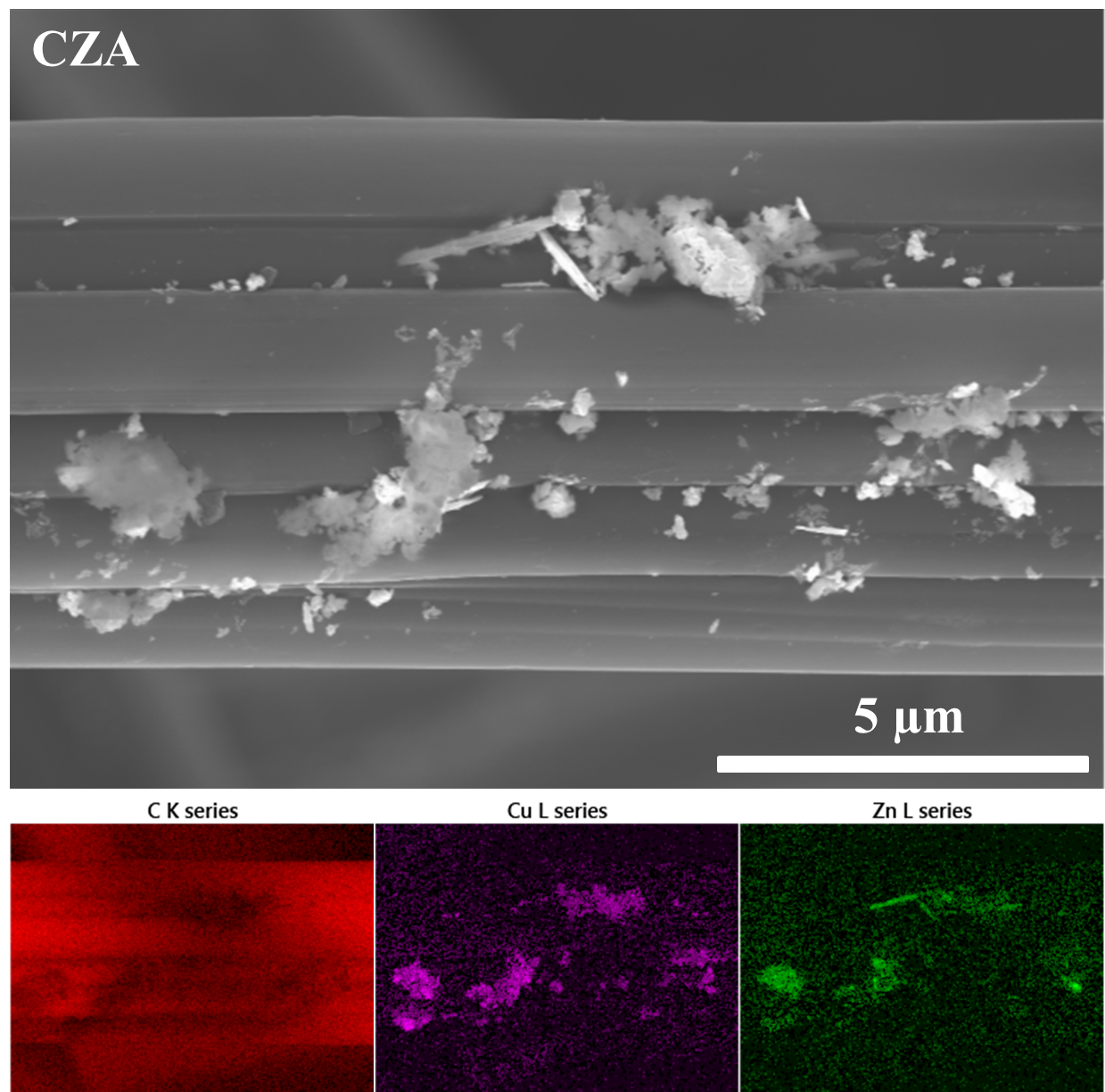


**Figure S2 |** EDS images of the CuO-ZnO combination structure with ACFs.

**Table S1** **|** Notations of the various metal oxide-doped ACFs.

|  |  | **Method** | | | **ICP** | |
| --- | --- | --- | --- | --- | --- | --- |
| **Sample** | **ACF**  **(g)** | **Zinc acetate dihydrate**  **(mmol/100 ml)** | **Sodium hydroxide**  **(mmol/100 ml)** | **Copper nitrate hydrate**  **(mmol/100 ml)** | **Zn**  **(wt%)** | **Cu**  **(wt%)** |
| **ZnO** | X | 500 | 500 | - | - | - |
| **ZnA-1** | 0.5 | 100 | 100 | - | 1.34 | - |
| **ZnA-2** | 0.5 | 250 | 250 | - | 3.02 | - |
| **ZnA-3** | 0.5 | 500 | 500 | - | 6.47 | - |
| **^*^CuA-3** | 0.5 | - | - | - | - | 7.31 |
| **CZA** | 0.5 | 500 | 500 | 50 | 5.33 | 8.28 |

*CuO-sputtered ACFs (Ref. Bai. BC, etc, Materials Chemistry and Physics 200 (2017) 361-367).
